# Complete alanine scanning of the *Escherichia coli* RbsB ribose binding protein reveals residues important for chemoreceptor signaling and periplasmic abundance

**DOI:** 10.1038/s41598-017-08035-5

**Published:** 2017-08-15

**Authors:** Artur Reimer, Vitali Maffenbeier, Manupriyam Dubey, Vladimir Sentchilo, Diogo Tavares, Manuel Hernandez Gil, Siham Beggah, Jan Roelof van der Meer

**Affiliations:** Department of Fundamental Microbiology, University of Lausanne, 1015 Lausanne, Switzerland

## Abstract

The *Escherichia coli* RbsB ribose binding protein has been used as a scaffold for predicting new ligand binding functions through *in silico* modeling, yet with limited success and reproducibility. In order to possibly improve the success of predictive modeling on RbsB, we study here the influence of individual residues on RbsB-mediated signaling in a near complete library of alanine-substituted RbsB mutants. Among a total of 232 tested mutants, we found 10 which no longer activated GFPmut2 reporter expression in *E. coli* from a ribose-RbsB hybrid receptor signaling chain, and 13 with significantly lower GFPmut2 induction than wild-type. Quantitative mass spectrometry abundance measurements of 25 mutants and wild-type RbsB in periplasmic space showed four categories of effects. Some (such as D89A) seem correctly produced and translocated but fail to be induced with ribose. Others (such as N190A) show lower induction probably as a result of less efficient production, folding and translocation. The third (such as N41A or K29A) have defects in both induction and abundance. The fourth category consists of semi-constitutive mutants with increased periplasmic abundance but maintenance of ribose induction. Our data show how RbsB modeling should include ligand-binding as well as folding, translocation and receptor binding.

## Introduction

Bacteria have been increasingly used as chassis for the construction of biosensors (“bioreporters” or “bactosensors”), which can be applied for easy measurements of toxic compounds in environmental samples^1–3^, of clinically relevant biomolecules^4^ or of metabolites in industrial processes^5^. In order to function in a biosensor the reporter cell is equipped with a genetic circuit encoding a sensory protein, which directly or indirectly alters transcription of a so-called reporter gene from a dedicated orthogonal promoter in presence of the molecular target^6–9^.

**Figure 1:**
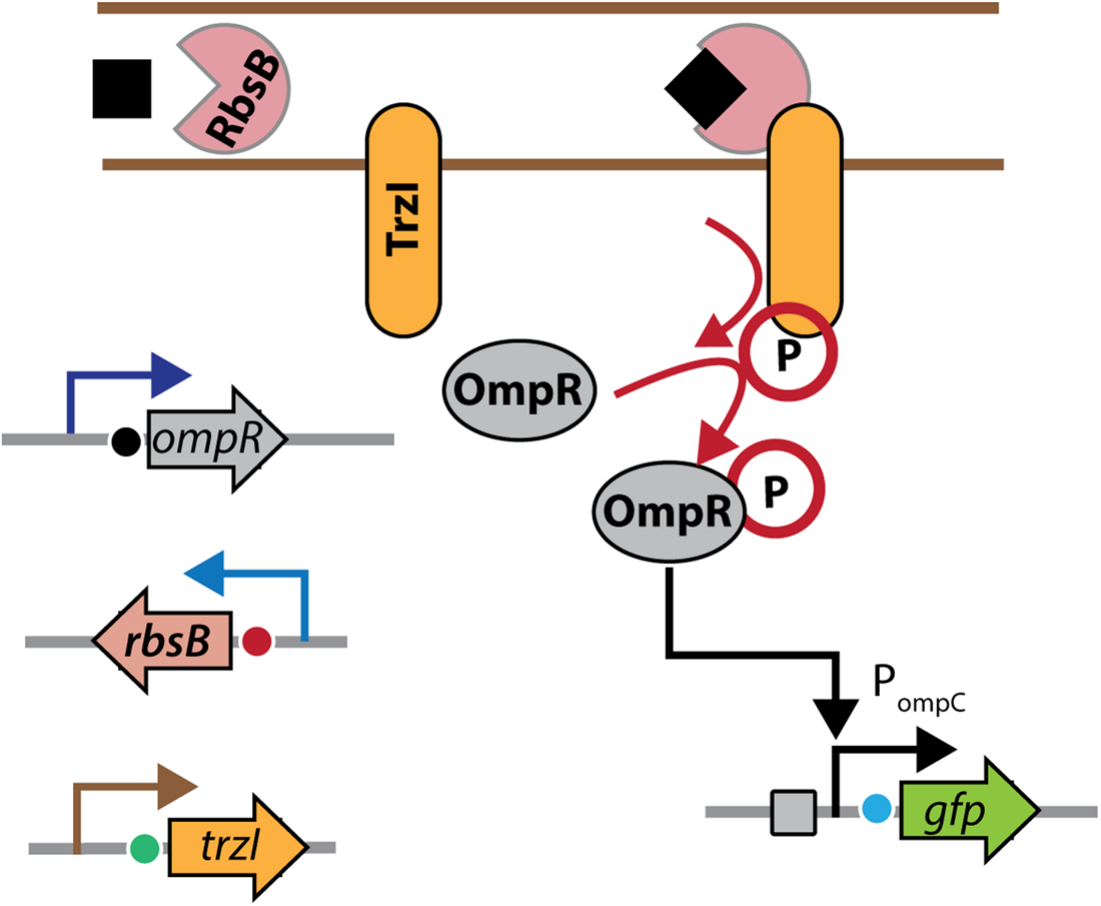
Schematic outline of the ribose-binding protein-based Gfpmut2 reporter signaling chain in *E. coli*. Ribose-binding protein (RbsB) captures its ligand ribose, leading to a conformational change. Ribose-RbsB binds the Trz1 hybrid transmembrane receptor, which causes a phosphorylation cascade leading to OmpR-P binding the *ompC*-promoter and consecutive *gfpmut2* expression.

One of the critical shortcomings of current bioreporters is the choice of sensory protein to operate the circuitry. Most bioreporters exploit naturally existing sensory proteins or transcription factors with their cognate target recognition properties^10^, but for many pollutants or compounds of interest no natural sensory proteins are known. Random mutagenesis of existing transcription factors followed by rigorous selection strategies have produced some mutants with interesting properties^11–13^, but this is a cumbersome approach that has to be optimized for each new protein scaffold. As an alternative for a potentially more universal mutagenesis and selection platform periplasmic binding proteins (PBPs) have been proposed. PBPs form a versatile superfamily of proteins, which due to their wide range of natural ligands and their abundance throughout different classes of microorganisms exhibit a good potential for protein engineering^14^. A seminal publication in 2003 by Looger *et al*. suggested the successful design of bioreporters based on PBPs with the help of computational protein prediction for specific detection of non-natural targets down to the nanomolar range^15^. Of special interest in bespoke publication was the design of a mutant of the RbsB ribose binding protein of *Escherichia coli* supposedly able to bind trinitrotoluene (TNT) with an affinity constant of 2 nM, and which, when expressed in *E. coli*, would elicit formation of beta-galactosidase from a hybrid signaling chain at concentrations as low as 0.1 nM TNT^15^. Despite the beauty of the concept, recent publications have been unable to reproduce the initial findings^16, 17^, and it has been suggested that the designed mutants resulted in unstable proteins^18^. In addition, it is likely that the initial computations concentrated solely on calculating new PBP binding site specificities on the basis of existing crystal structures with or without bound ligand, but neglecting the other characteristics of a functional PBP. This may include processes such as: folding and translocation into the periplasm, dynamic ligand-binding (cleft opening and closing), and productive interactions of ligand-bound PBP to its membrane channel or receptor^19–23^. Possibly, computational design of binding specificities of dynamic proteins such as PBPs can be improved with better scoring functions that embed empirical data on each step of its functional cascade. As a start to better functionally characterize the importance of amino acid residues of RbsB in the complete ribose-induced signaling chain, we carried out an alanine scan of the near complete protein. Results from a limited alanine scan of RbsB have been published previously^24^. To proceed, a total of 235 RbsB mutants were produced by DNA synthesis in which the original residue was replaced by Ala. These mutants were individually expressed in an *E. coli* chassis with the Trz1 hybrid signaling pathway between the periplasmic part of the Trg chemoreceptor and the cytoplasmic part of EnvZ, enabling production of the GFPmut2 fluorescent protein^16, 25^ in response to ribose addition (Fig. 1). GFPmut2 expression was analyzed by flow cytometry in cells exposed or not to ribose. Additionally, RbsB levels in the periplasmic space of *E. coli* were quantified by nano liquid chromatography followed by direct peptide mass identification (LC-MS) in a selection of 25 mutants with the strongest defects plus the wild-type. Our results highlight a variety of critical amino acid residues for ribose-induced RbsB signaling also at seemingly inconspicuous positions, which help to understand previous irreproducible design results.

## Results

### Production of an Ala-substitution library of RbsB

A near-complete Ala-substitution library of the *E. coli* RbsB protein was produced from a combination of pooled mutant DNA synthesis and individual mutant recovery or construction. Despite multiple repeated attempts, three replacements could not be obtained (i.e., 135T, 136S and 145F). 37 positions (14%) of RbsB wild-type contain an Ala-residue and were not further changed in this work. All mutant *rbsB* genes were transferred to the generic vector pSTVP_AA_mcs for expression from the constitutive P_AA_-promoter^16^, and verified by sequencing again after final cloning. Noteworthy, several Ala-replacements in RbsB led to appreciable slower growth of the *E. coli* expression strain, apparent as smaller colonies on plates and lower counts in flow cytometry analysis (Fig. S1).

### Effect of Ala-substitution on inducibility with ribose

**Figure 2:**
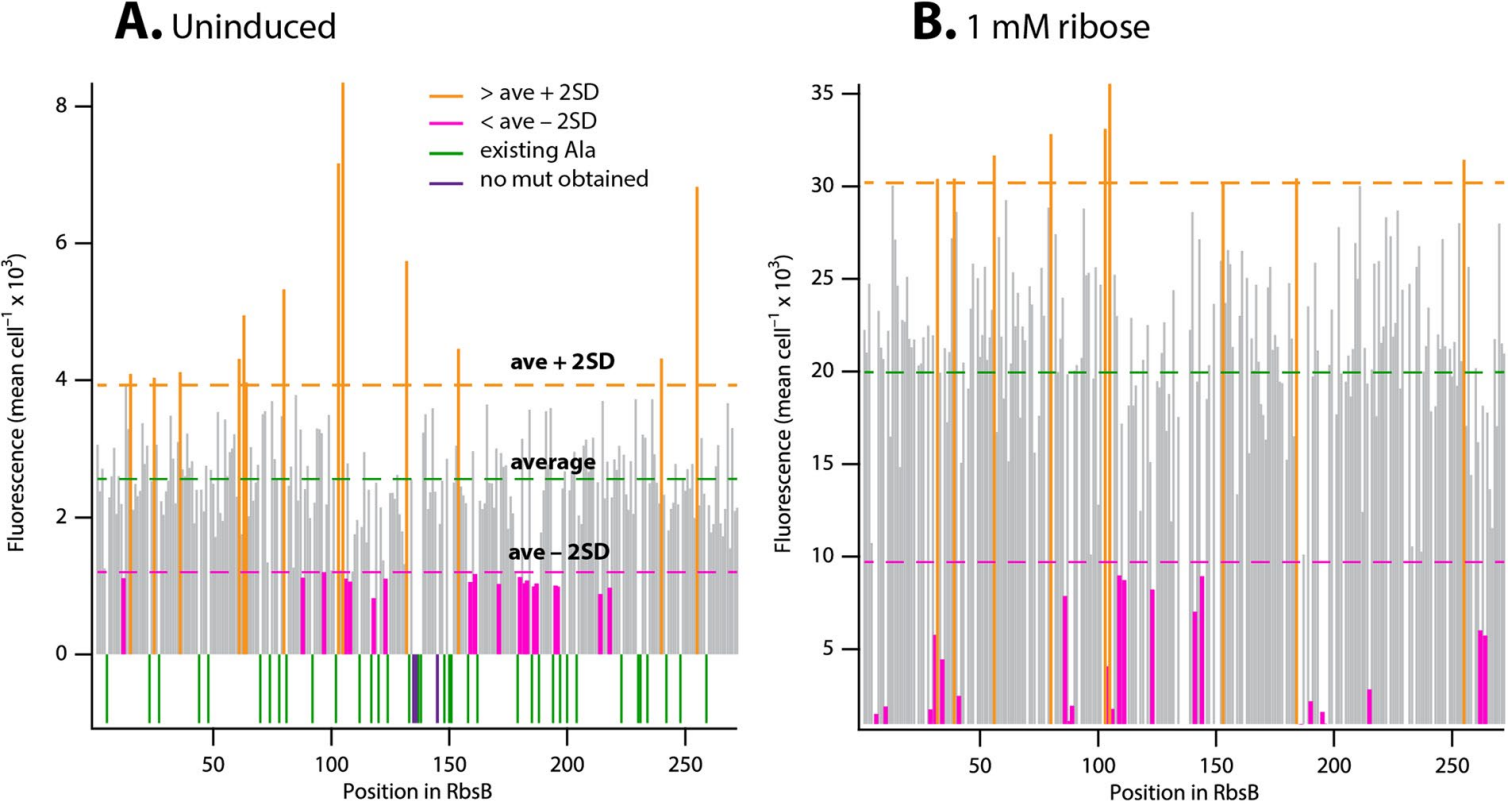
GFPMut2 average fluorescence values from the *E. coli* Trz1-OmpR, *ompCp-gfpmut2* signaling chain for each RbsB mutant, sorted by position. (**A**) Values for uninduced conditions (i.e., absence of ribose). (**B**) Values after 2 h induction with 1 mM ribose. Stacks represent the average cell fluorescence in flow cytometry from independent biological triplicates. Values in orange are those higher than the average across all mutants and wild-type + 2 × *SD* (considered semi-constitutives), those in magenta are lower than the average −2 × *SD* (considered poorly or non-inducible). Negative stack values in panel A in green denote existing RbsB wild-type Ala-positions; those in purple represent the three positions for which no substitutions could be obtained (135T, 136S and 145F).

The final 232 mutants were analyzed in triplicate assays by flow cytometry for GFPMut2 fluorescence as a result of induction by ribose from the hybrid Trz1-OmpR-*ompCp-gfpmut2* signaling chain (Fig. 1), in comparison to *E. coli* expressing wild-type RbsB (strain 4175) as well as *E. coli* without *rbsB* (strain 4497) (Dataset 1). Inducibility with 1 mM ribose was measured on cultures grown and exposed in 96-well plates on different occasions; therefore, every plate contained a separate triplicate wild-type positive and a negative control. In general terms, we observed both partial and complete loss of inducibility by ribose, appearance of semiconstitutives (with higher values of GFPMut2 fluorescence than wild-type in cells not exposed to ribose), as well as similar inducibility as wild-type RbsB (Fig. 2 and Dataset 1). Ten percent of Ala-substitutions led to a loss of ribose induction, which we defined from a fluorescence less than the average minus two standard deviations (*SD*), calculated from the data variation among all mutants and wild-type (Fig. 2B and Dataset 1). Among those, half were not inducible at all, or even showed lower fluorescence in presence than in absence of ribose. Approximately, seven percent led to a statistically significant increased GFPMut2 fluorescence (i.e., higher than the average plus two *SD*) in absence of ribose compared to wild-type RbsB (Fig. 2A and Dataset 1). Ribose induction profiles allowed to group the Ala-replacement mutants of RbsB loosely into three broad groups shown in Table 1, based on the criteria described above (e.g., no or weak induction, semi-constitutive).

**Table 1:** Ala-replacement mutants of RbsB causing significant change in ribose induction.

Mutant nature	Position	Amino acid	GFPMut2 Fluorescence		Fold induction^b^
			Uninduced	Induced^a^	
Wild-type	—	—	2851 ± 544	23663 ± 2958	8.30
Non-inducible^c^	**6** ^d^	L	2293 ± 107	1495 ± 34	0.65
	**10**	T	2604 ± 629	1884 ± 454	0.72
	**215**	D	3696 ± 40	2816 ± 25	0.76
	**89**	D	2416 ± 503	1932 ± 295	0.80
	**29**	K	2039 ± 49	1736 ± 38	0.85
	**41**	N	2823 ± 409	2459 ± 84	0.87
	**190**	N	2396 ± 39	2179 ± 92	0.91
	**186**	V	992 ± 133	940 ± 119	0.95
	**196**	G	990 ± 155	950 ± 103	0.96
	**88**	L	1123 ± 55	1103 ± 28	0.98
Poorly inducible^e^	**195**	L	1005 ± 37	1594 ± 51	1.59
	**106**	V	1107 ± 262	1761 ± 262	1.59
	**264**	D	3059 ± 161	5726 ± 456	1.87
	**34**	L	2207 ± 229	4432 ± 115	2.01
	**31**	G	2537 ± 343	5761 ± 165	2.27
	**262**	P	1906 ± 418	6004 ± 1649	3.15
	104	D	1235 ± 34	4068 ± 596	3.29
	**141**	R	2123 ± 494	7006 ± 1474	3.30
	**86**	I	2246 ± 260	7873 ± 2393	3.51
	**144**	G	2378 ± 89	8926 ± 348	3.75
	**111**	I	1963 ± 170	8728 ± 590	4.45
	**237**	P	2923 ± 244	10267 ± 2976	3.51
	**233**	I	3166 ± 217	10555 ± 2856	3.33
Semi-constitutives^f^	**105**	N	8344 ± 171	35539 ± 219	4.26
	103	S	7167 ± 167	33101 ± 1963	4.62
	255	E	6824 ± 324	31441 ± 595	4.61
	**132**	I	5741 ± 731	24383 ± 145	4.25
	80	Q	5324 ± 203	32831 ± 1549	6.17
	**63**	I	4945 ± 134	20414 ± 660	4.13
	154	F	4460 ± 102	23688 ± 340	5.31
	240	I	4314 ± 395	23434 ± 3754	5.43

^a^Average cell fluorescence in flow cytometry after 2 h induction with 1 mM ribose, averaged from biological triplicates ± SD. ^b^Fold-induction is the ratio of the average cell fluorescence with ribose (induced) and without ribose (uninduced). ^c^Fold-induction at or below 1 in presence of 1 mM ribose. ^d^Positions in bold type-face indicate mutants whose expression in the periplasmic space was verified by quantitative peptide mass spectrometry. ^e^Induced values lower than the average induction across all mutants including wild-type, minus 2× the SD across all mutant induction data. ^f^Uninduced values minus their SD higher than the average uninduced value across all mutants including wild-type, plus 2× the SD across all mutant uninduced data.

### RbsB mutant periplasmic space abundance

Since some of the differences in ribose induction compared to wild-type RbsB may be due to differences in RbsB-mutant stability, folding and/or translocation to the periplasmic space, we analyzed periplasmic protein abundance for a selection of 25 mutants with the strongest defects from the groups in Table 1, and of wild-type, grown under standard conditions as for ribose induction. Analysis of SDS-PAGE separated periplasmic protein fraction in the range of 28–40 kDa, trypsin-digested and quantified by nano LC-MS, revealed a total of 424 distinct proteins (Fig. S2). RbsB (mutant) peptides were identified and quantified from unique fragments (exclusive spectrum count), normalized by total spectrum count per sample and further by *D*-galactose-binding periplasmic protein (MglB) counts as internal standard (Table 2). Samples with a significantly lower abundance of RbsB mutant peptides than expected from variation of the MglB internal standard (F-test; p < 0.05) were considered carrying a mutation affecting folding, translocation and/or stability of the mutant RbsB in(to) the *E. coli* periplasm (Table 3). Assuming proportional decrease of GFPmut2 expression and the normalized RbsB periplasmic abundance (Table 3), we conclude that Ala-substitutions at D89, V106, I111, R141, G144, V186, G196, D215, P262, and D264 primarily have signaling defects, since their abundance is similar (or even slightly higher) as wild-type, but GFPmut2 fluorescence upon induction with ribose is lower (Table 3). In contrast, Ala-substitutions at I63, I86, N190 and I233 primarily seem to affect protein synthesis, folding, stability and/or translocation, since their fluorescence decreases (except for I63, a semi-constitutive) as much as their abundance in comparison to wild-type RbsB (Table 3). Ala-substitutions in L6, T10, K29, G31, L34, L88, N41 and L195 seem to affect both processes, since the loss in abundance is not sufficient to explain the decrease in normalized fluorescence through the signaling chain (again assuming proportionality between the two, Table 3). Of note that MglB abundance in mutants L6A, V106A and V186A itself was lower than expected from the variance across all samples (Table 3). Therefore, these RbsB mutants may fold to such a state that they block general protein translocation channels for other periplasmic binding proteins. Mutants P237A and I111A showed higher than expected abundance but lower normalized fluorescence and, therefore, are also impaired in signaling. Finally, mutants I132A and N105A showed a higher level of periplasmic abundance than expected from wild-type RbsB, but only N105A expressed constitutively higher GFPmut2 fluorescence in absence of ribose (Table 1).

**Table 2:** Exclusive and normalized mass-spectrometry abundance of periplasmic RbsB or selected alanine-replacement mutants compared to the internal standard protein MglB.

Strain expressing RbsB or RbsB mutant	Abundance				
	Exclusive spectrum count^b^		Normalized on total spectra^c^		Normalized to MglB^d^
	RbsB or RbsBmut	MglB	RbsB or RbsBmut	MglB	RbsB or RbsBmut
K29A	9	36	10	38	**10** ^e^
L34A	20	43	20	44	**17**
G31A	18	40	20	44	**17**
N41A	8	45	8	43	**7**
I63A	13	24	16	29	**21**
I86A	18	45	17	43	**15**
D89A	37	32	33	29	43
N105A	61	35	60	34	67
I111A	29	14	37	18	78
I132A	63	34	58	31	71
R141A	43	38	37	33	43
G144A	27	33	27	33	31
N190A	5	53	5	54	**4**
D215A	62	46	65	48	51
I233A	24	54	24	53	**17**
P237A	70	33	59	28	80
P262A	51	32	49	31	60
D264A	69	41	73	43	65
RbsB	50	41	57	46	47
RbsB(2)	115	46	134	54	95
L6A	35	0	47	**0**	**NC** ^**f**^
T10A	13	31	10	23	**16**
L88A	38	47	47	59	**31**
V106A	65	11	71	**12**	224
V186A	27	13	18	**8**	79
L195A	31	24	31	24	**49**
G196A	36	18	45	22	**76**

^a^D-galactose-binding periplasmic protein (MglB) was used as internal standard for RbsB quantification based on similar molecular weight and abundance. ^b^Absolute number of detected peptides exclusive to the protein. ^c^Number of peptides exclusively identifying the protein, normalized to the same total number of identified peptides in each sample. ^d^Normalized values to the average normalized total spectra values of MglB. ^e^Values in bold indicate periplasmic samples with statistically significantly (F-test; p < 0.05) lower abundance than wild-type RbsB as expected from the variance of MglB. ^f^NC, no correction because of absence of MglB in periplasm.

**Table 3:** Combined deduced effects of selected RbsB Ala-substitution mutants.

Effect	Position	Induced fluorescence^a^	MS expression^b^	Normalized fluorescence^c^	Normalized abundance^d^
Wild-type	—	23663	47	1	1
Signaling and abundance	L6	*1495*	**(23)**	0.063	NC^e^
	T10	*1884*	**(8)**	0.080	(0.168)
	K29	*1736*	**10**	0.073	0.212
	G31	*5761*	**17**	0.243	0.367
	L34	*4432*	**17**	0.187	0.367
	N41	*2459*	**7**	0.104	0.150
	L88	*1103*	**(15)**	0.047	0.323
	L195	*1594*	**(24)**	0.067	0.512
Abundance	I63	20414	**21**	0.863	0.445
	I86	*7873*	**15**	0.333	0.319
	N190	*2179*	**4**	0.092	0.075
	I233	10555	**17**	0.446	0.365
Signaling	V106	*1761*	(111)	0.074	2.36
	P262	*6004*	60	0.254	1.276
	G196	*950*	(38)	0.040	0.800
	V186	*940*	(39)	0.040	0.831
	D264	*5726*	65	0.242	1.370
	D215	*2816*	51	0.119	1.093
	G144	*8926*	31	0.377	0.660
	R141	*7006*	43	0.296	0.905
	I111	*8728*	78	0.369	1.659
	D89	*1932*	43	0.082	0.918
	P237	10267	80	0.434	1.701
No effect	I132	24383	71	1.030	1.510
	N105	35539	67	1.502	1.424

^a^Average GFPmut2 fluorescence of cells induced for 2 h with 1 mM ribose (Table 1). Values in italics are less than the average minus 2 × *SD*. ^b^Normalized mass spectrometry abundance (Table 2). Values in bold type-face are those with statistically significantly lower abundance than expected from the observed variance. Values in brackets are further normalized for ease of comparison to the RbsB wild-type level of 47 (Table 2). ^c^Ratio of GFPmut2 fluorescence in mutant versus wild-type RbsB. ^d^Ratio of normalized RbsB-mut peptide mass count to wild-type RbsB. 99% confidence interval: 0.785–1.274. ^e^NC, not corrected because of absence of measurable MglB in the periplasm.

### Positions of Ala substitution effects

**Figure 3:**
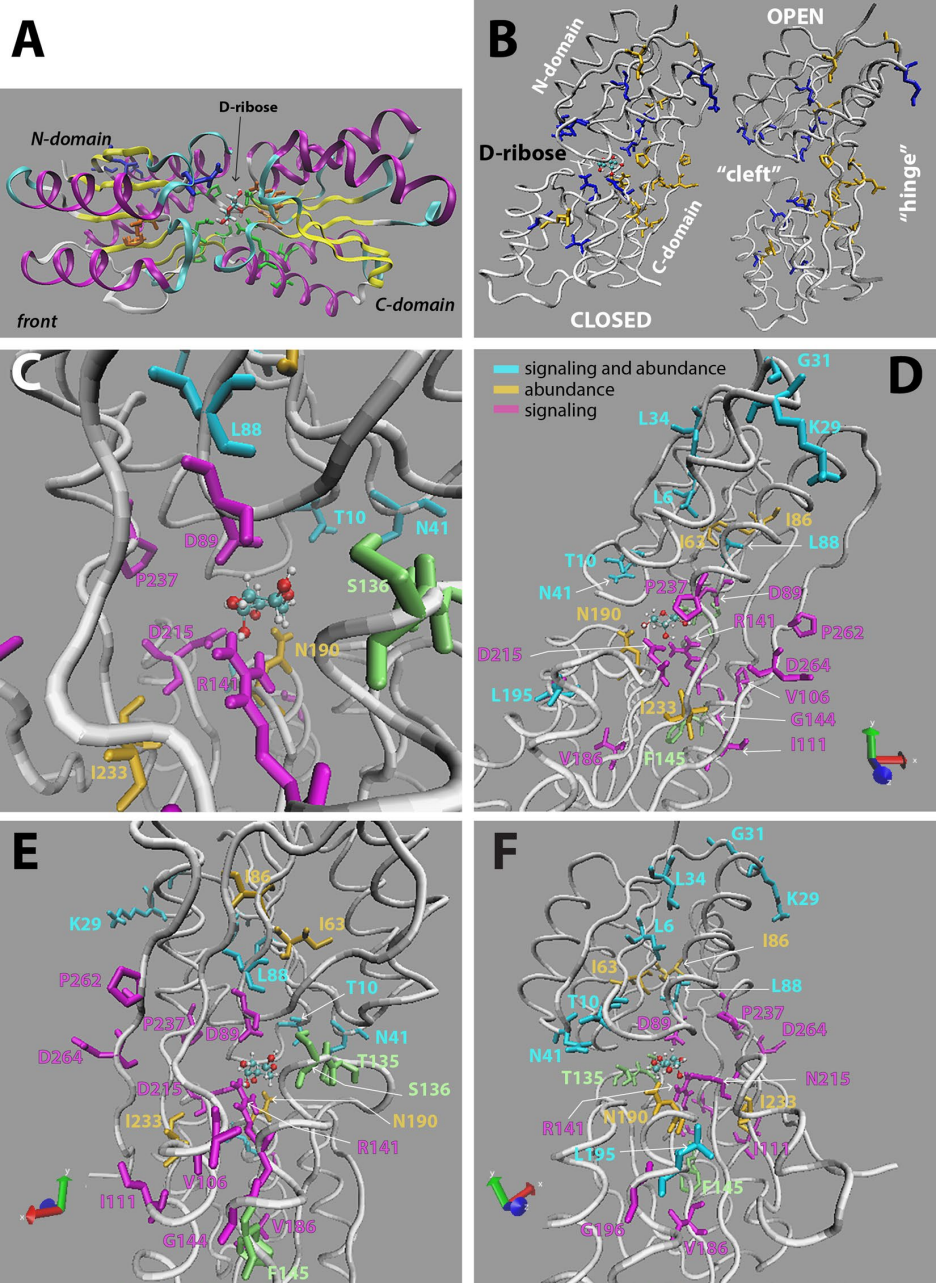
Positions of the Ala-substitution mutants with the strongest observed effects on ribose induction. (**A**) Crystal structure of closed RbsB with ribose in pyranoside form (PDB: 2DRI), alpha-helices in purple, beta-sheets in yellow and turns in cyan. Ribose is placed as licorice-structure. N- and C-domains, nomenclature according to ref. 33. (**B**) Side-view comparison of closed and open conformation RbsB (PDB: 1URP, chain A), with “hinge” region and ligand-“cleft” indicated. Residues, whose substitution by Ala caused complete loss of induction by ribose in blue; those with lower induction in gold. (**C**) Detail of the ribose binding pocket in the closed form of RbsB. Cyan: residues affecting periplasmic abundance and signaling; magenta, affecting signaling only; gold, affecting abundance; green, residues for which no Ala-replacement mutant could be obtained. (**D**–**F**) Positions of the various mutants and their categorization on the RbsB closed conformation in different rotation views. Top part is always the N-domain. Image panels produced using VMD visual molecular dynamics editor^32^.

The Ala-substitution mutants with observed effects cover any part of RbsB except a few helices (Fig. 3A,B). Not surprisingly, several mutants are located within the ribose binding pocket, notably D89A, N190A, D215A and R141A, and to a lesser extent L88A (Fig. 3C). At the cleft entry are two mutants T10A and N41A. On the back side of the binding pocket is the flexible hinge region (Fig. 3B) where six of the mutants can be found. These are D104A, V106A, I233A, P237A, P262A and D264A (Fig. 3D). Furthermore, two mutations causing a semi-constitutive phenotype (S103A and N105A) are also positioned in the hinge region. Several more mutants locate in the N-domain of RbsB (Fig. 3B); notably in a peripheral corner in a helix on the “top” (K29A, G31A) and within beta-sheets (L6A, L34A, I86A, Fig. 3D–F). Furthermore, prominent arms in the C-domain display two groups of consecutive mutants (G144A, which is close to the R141 in the binding site; and L195A, G196A and V186A in the arm supporting the N190 binding site residue, Fig. 3D–F). Interestingly, two of the positions for which no substitutions could be obtained (T135, S136) locate on the edges of the cleft of RbsB in the structure (Fig. 3E), which may therefore be also critical for its folding and functioning.

Several of the critical positions we found here are not absolutely conserved among members of the cd06323:PBP1_ribose_binding-domain containing family (Fig. S3). Interestingly, the third missing Ala-replacement mutant (F145) is at a conserved position, as is its neighbor G144 (G144A caused semi-constitutive expression). Vice-versa, many of the conserved amino acids in this taxonomically extremely widespread domain family can be substituted in RbsB by Ala without noticeable effects (Fig. S4, hash-signs).

## Discussion

One of the major hurdles in bacterial bioreporter development is the choice of the chemical target receptor protein. Whereas most current bioreporters have exploited naturally existing transcription regulators or sensory proteins with their cognate ligand specificities^26^, for detection of new (often manmade) molecules it will be necessary to find or engineer new ligand-binding properties^27^. One of the proposed possibilities for engineering new ligand-binding properties has been the periplasmic-binding-protein scaffold, for which crystal structures in absence and presence of ligands are known^14, 21, 22, 25^. Predictions and experimental data from a decade ago suggested that completely new ligand specificities could be obtained on scaffolds such as the ribose-binding protein (RbsB) of *E. coli*, changing, for example, from ribose to trinitrotoluene^15^. Key results from this work could not be reproduced and have been seriously challenged^16^. It thus appears that our knowledge on the various steps of RbsB-ligand signaling, such as binding of the ligand and binding of the ligand-protein complex to the receptor, as well as RbsB folding, periplasmic translocation and stability itself, must be as yet incomplete. Hence, attempts for prediction of new ligand-binding specificities solely on the basis of modeling of the PBP binding pockets may lead to false results.

Here we focused on obtaining a better understanding of the influence of the individual RbsB residues on ligand-induced signaling as well as RbsB periplasmic space abundance. A near complete series of Ala-substitution mutants of non-Ala residues of RbsB was produced and characterized for loss of ribose-induced signaling in *E. coli* (Table 1). A total of 23 mutants was found, which resulted in stronger decrease than expected by variation of GFPmut2 expression from the Trz1-OmpR hybrid signaling pathway upon ribose induction. Several other mutations were found that resulted in a significant increase of fluorescence signal irrespective of ribose addition. Mutants displaying significant defects in GFPmut2 induction were further examined for RbsB-mutant protein abundance in the periplasmic space (Table 2). From the combined results we can infer whether an Ala-substitution acts on the signaling pathway itself (hence: ligand and/or receptor binding), on protein abundance (assumed to be the result of defects in either translation, folding and/or translocation, or stability), or both. Our assumption here was that a decrease in periplasmic abundance might lead to a proportionally diminished fluorescence reporter output from the signaling chain, although this relation may be more complex for individual RbsB mutants.

First we want to highlight the presumed ribose binding pocket, which has already been subject of a previous investigation by Vercillo *et al*. who constructed 13 alanine-substitution mutants in residues on the basis of their proximity to bound ribose in the crystal structure^24^. In the ribose-binding pocket of RbsB we found five mutants leading to a defect in the behavior of the bioreporter (Fig. 3C). Mutants D89A, D215A, R141A, and N190A showed a strong loss of inducibility, but the periplasmic abundance of the N190A mutant was proportionally as low as the loss in ribose-induced GFPmut2 fluorescence. This suggests that N190 is not so much involved in ribose binding but a key residue influencing protein folding, stability and/or translocation into the periplasm. Vercillo *et al*.^24^ demonstrated that Ala-substutions at positions 89, 215, and 190, cause loss of ribose binding by the purified protein. Purified N190A mutant protein displayed a significant decrease in stability^24^, which is in agreement with our findings. R141A was not part of their mutant selection. In contrast, we did not find statistically significant effects at 1 mM ribose induction of Ala-substitutions on F16, F164, F214 and Q235 as well as N64, which were previously reported as losing ribose binding capacity^24^.

Mutants N13A and F15A, which are located close to the entrance of the cleft, displayed a semi-constitutive behavior, similar to I132A and N105A (Table 1 and Dataset 1). Due to their location, N13A and F15A might change the ratio between the open and closed conformation of the protein, and, in this way, lead to constant recognition by the Trz1 chemoreceptor and thus higher background fluorescence. According to Vercillo *et al*. the purified N13A and F15A mutant proteins (in an L265C RbsB background) are stable and bind ribose, albeit (slightly) diminished compared to wild-type^24^. This may explain their semi-constitutive behavior in our reporter background. Mutation of the nearby residue I132, located at the entrance of the pocket, produced an even stronger semi-constitutive phenotype as N13A or F15A, which is in agreement with previous results that purified I132A has similar stability and ribose-binding as wild-type RbsB^24^. Position I132 had also been shown by Björkman *et al*. to play a crucial role in the intra-protein connection between the two domains of RbsB (Fig. 3B) in order to facilitate the closed conformation^22^. The connection partner for the formation of the van der Waals contact is position N41^22^. An alanine substitution at this place led to a strong growth defect (Fig. S1), a significant decrease of the periplasmic abundance of the protein and a complete loss of induction (Tables 2 and 3). We therefore conclude that this position is both important for protein stability and for ribose- or Trz1 binding (Fig. 3F). Notably, also two residues for which no substitution could be obtained, locate in the entry cleft (T135, S136, Fig. 3E,F).

On the opposite side of the ligand entrance is the hinge region (Fig. 3B). Here we detected two groups of mutation effects, which may therefore influence RbsB’s ability for adopting conformational changes. The first group, consisting of D104A, V106A, I233A, P237A, P262A and D264A, led to a significant loss of induction by ribose, which became more dramatic the closer the residue’s location to the hinge (Tables 1 and 3, Fig. 3D,E). The other group, with S103A and N105A, resulted in opposite, more constitutive behavior, but with maintenance of induction by ribose (Table 1). Four of the mutants in this region, D264A, P262A, P237A and N105A, even displayed higher periplasmic abundance than wild-type RbsB (Tables 2 and 3), suggesting a decreased cytoplasmic pre-folding and thus a potentially higher secretion rate^28^. Although we did not specifically examine S103A’s abundance in the periplasm, we assume it to be similar to N105A because of its high fluorescence value (Table 1). The higher background of S103A and N105A mutations, but maintenance of ribose induction potential may be due to more limited hinge movement but adoption of a configuration favorable for contact to the receptor. This would be in agreement with the measured wild-type stability and similar wild-type binding constant of ribose for purified S103A protein^24^. In contrast, the effect of the mutants D264A, P262A and P237A may be the result of an unfavorable change to the hinge region, which prevents adoption of the closed form upon binding of ribose or impedes subsequent contacts to the Trz1 receptor (none of these mutations have been previously analyzed).

Mutants L6A, L34A, I86A, and I63A locate within the two β-sheets in the interior of the protein N-domain (Fig. 3D,E) and showed a significantly reduced periplasmic abundance, suggesting a strong influence on RbsB folding, stability or translocation. The loss of ribose-induced signaling for L6A and L34A was proportionally stronger than can be explained from their decreased periplasmic abundance. Therefore, this position may have an additional influence on the signaling process. Interestingly, strains expressing L34A also grew poorer (as N41A), which may point to these proteins clogging *E. coli*’s translocation systems^28^. Surprisingly, mutant I63A showed a semi-constitutive response despite a lower periplasmic abundance. Noteworthy, the strain expressing mutant L6A showed a complete absence of MglB (galactose-binding protein) in its periplasm, in comparison to the other strains (Table 3). This mutant may thus cause extreme folding defects that inhibit translocation of other PBPs. The location of many Ala-replacement mutants with lower RbsB periplasmic space abundance in the N-domain is in agreement with the location of most spontaneous RbsB suppressor mutants that overcame a defect in SecB-dependent translocation^19^. It suggests that the N-domain (as that part of the protein that passes first through the translocation channel) is particularly sensitive to misfolding.

Positions I111, G144 and F145 are situated at two alpha-helices of the C-terminal part (Fig. 3E). Ala-substitutions in two of these residues caused a significant loss of induction, which could not (I111A) or only partially (G144A) be explained by reduced periplasmic abundance. Mutant F145A could not be obtained, despite multiple trials. This is a conserved residue among a wide range of periplasmic-binding proteins of the cd06323:PBP1_ribose_binding protein domain family, suggesting it plays a pivotal role (Fig. S3). Similarly, substitutions in L195, G196, and V186 in other helices and sheets in the C-domain caused drastic effects on induction and/or on protein abundance (L195A, Table 3). This suggests some global structural change of the protein resulting in a perturbation of the hinge region and thus influencing the relative positions of the residues on the cleft side, similar as has previously been suggested on an analysis of the RbsB mutant I111R^23^.

**Figure 4:**
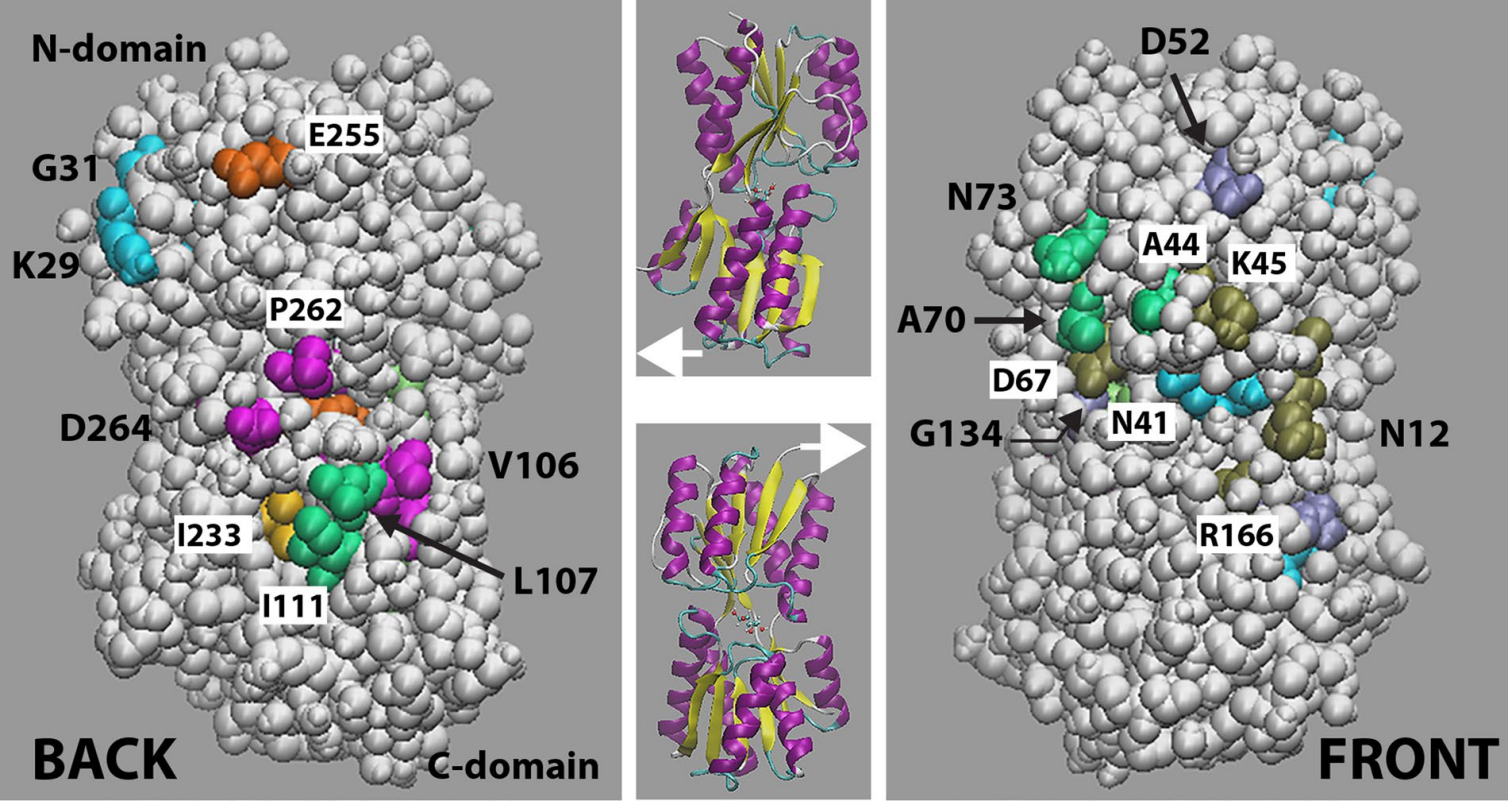
Surface exposure of various mutant classes positioned on RbsB in closed conformation with ribose, here shown as Van der Waals-atom surface including water molecules (PDB: 2DRI). Secondary structure cartoons indicate the positions of protein in FRONT and BACK views. Color code: cyan (K29, G31, N41), residues identified here that affect protein abundance and signaling; magenta (V106, P262, D264), residues affecting signaling; gold (I233), residue affecting protein abundance only; orange (E255, S103 - just below P262), Ala-substitutions causing more constitutive signaling; green (A44, A70, N73, I111, L107), residues affecting chemotactic receptor and ribose transport channel binding^23^; tan (N12, K45, D67), residues affecting ribose transport^23^; iceblue (D52, G134, R166), residues affecting contact to ribose transport channel^20^. Image panels produced using VMD visual molecular dynamics editor^32^.

One would expect to have produced substitutions causing a defect in ribose-bound-RbsB binding to the Trz1 receptor, leading to decreased signaling and, hence, decreased Gfpmut2 fluorescence. Amidst the list and location of mutants causing effects that cannot be more simply explained by mechanistic details of ribose binding or hinge-movement, or residues of structural importance (e.g., Gly- or Pro-residues), we find two conspicuous mutations: K29A and E255A. Both of these locate to the left outside part of the protein’s N-domain (Fig. 4), but whereas substitution in K29A abolishes induction, that in E255A causes a semi-constitutive phenotype (Dataset 1 and Table 1). Both residues are charged, locate on the outside (Fig. 4) and change their relative orientation between open and closed configuration (Fig. 3B). On the other hand, these residues have not been detected in previous spontaneous or constructed RbsB mutants with inferred receptor or permease-binding defects^19–23^. The RbsB region contacting Trz1 has not been characterized, but, according to previous work analyzing RbsB mutants impaired in ribose chemotaxis, it would cover residues I111 and L107 on the “back” of the protein (Fig. 4) and A44, A70, N73 and G134 on the “front” (Fig. 4)^23^. Part of these residues (A44, A70, N73, I111, L107) overlap with the proposed region on RbsB binding the ribose permease^20, 22, 23, 29^ (Fig. 4). None of the Ala-substitutions to those residues (Fig. 4), except I111A, caused measurable loss of Trz1 signaling in our experiments, but D52A and R166A showed elevated background expression (Dataset 1), which may be due indirectly to altered ribose transport^20^.

Our study largely expands previous more limited mutation analyses of RbsB^20, 23, 24^, resulting in a more complete view on the importance of RbsB residues for protein stability and transport, as well as for proper signaling in the Trz1 transduction pathway. In addition to the more obvious residues implicated in ribose binding, we were able to highlight the importance of various other inconspicuous residues effecting the folding, structure or transport of the protein and also the signal transduction towards the chemoreceptor. Several of the positions we identified as crucial for proper RbsB functioning had been in fact targeted by the aforementioned RbsB protein design study, such as D89(S), R141(S), N190(F) or D215(S)^15^. Although it is possible that the negative effect of an Ala-substitution is absent in another substitution, this suggests that great caution has to be taken when targeting residues that are critical for ligand binding (D89, R141, D215) or protein stability and transport (N190). On the other hand, it suggests that mutant ligand-binding RbsB with poor structural stability may be improved by further mutating residues implicated in folding or translocation, the importance of which was shown here. We expect that our results may help future computational design of RbsB and PBPs more in general, by improving the scoring functions used in the force field calculations.

## Methods

### Strains and growth conditions

All *E. coli* strains used for this work are listed in Supplementary Table 1. For cloning purposes, *E. coli* strains were cultured at 37 °C on Luria Bertani (LB) medium^30^, supplemented with appropriate antibiotics to select for plasmid maintenance. In case of ampicillin (Amp), a concentration of 100 µg ml^−1^ was used; for chloramphenicol (Cm), we used 30 µg ml^−1^. Culturing conditions for protein overexpression and for reporter assays are specified below.

### Library construction

The mutant library was produced by DNA synthesis (DNA2.0, CA, USA) on the basis of the sequence provided in Fig. S4. The gene sequence further encoded a C-terminal hexahistidine (His_6_) tag framed by MfeI sites, restriction sites for NdeI (N-terminal), SalI (C-terminal), and for NcoI at the end of the signal sequence (Fig. S4). In first instance, the pooled DNA synthesis product was digested with NdeI and SalI and ligated into vector pSTV28P_AA_mcs, cut with the same enzymes (Fig. S5). This reporter construct library was cotransformed with pSYK1 into *E. coli* BW25113Δ*rbsB* ^16^. The transformants were plated and 800 single colonies were picked, verified by sequencing and organized in a 96-well format. This screening covered 74% of the mutants in the library. Further mutants were then recovered individually from the original synthesized templates by PCR, which were cloned into pSTV28P_AA_mcs, cotransformed, verified and stocked as before. A total of 17 mutants which could not be recovered from the synthesized mutant library were produced individually using the DpnI method^31^. Plasmid pAR3 (with wild-type *rbsB* in pSTV28P_AA_mcs; ref. 16) was amplified in the PCR using overlapping but reverse complementary primers with point mutations to create Ala-replacements at the desired codons in RbsB. The PCR product was then digested by DpnI at 37 °C for 1 h to remove exclusively the (methylated) template plasmid DNA. After inactivation of the enzyme at 80 °C for 20 min the PCR product was transformed in *E. coli* DH5a and transformants were selected on LB with Cm. Ten transformants were verified by sequencing of the re-amplified *rbsB-*insert for the presence of the intended mutation in *rbsB*. If correct, the insert was recovered by restriction enzyme digestion and ligated to pSTV28P_AA_mcs. Those constructs were again verified by sequencing and if correct, the mutant plasmids were cotransformed into *E. coli* BW25113Δ*rbsB* together with pSYK1 as before.

### RBP-based bioreporter assays using the Trz1-OmpR hybrid signaling chain

In order to measure the capacity of RbsB or its alanine-mutants to induce the Trz1-hybrid-OmpR *ompCp-gfpmut2* signaling chain in the presence of ribose, we used *E. coli* BW25113Δ*rbsB* cotransformed with pSTV-based plasmids (pAR3-K1A – pAR3-L272A for the alanine mutants, or pAR3, for wild-type *rbsB*) and plasmid pSYK1 (to provide the hybrid signaling chain)^16^. Upon induction, these strains produce GFPmut2, the fluorescence intensity of which was measured using flow cytometry. The bioreporter assay was optimized for minimal background GFPmut2 expression and medium fluorescence, according to ref. 16. Strains were inoculated in 96 well plates (F96 Cert.Maxisorp, Nunc, Denmark) containing 200 µL of minimal medium with Amp and Cm^16^ and 20 mM fumarate as sole carbon and energy source, with cells from organized stocks stored at −80 °C. Cultures were incubated overnight at 37 °C with rotary shaking at 700 rpm. The next morning, a volume of 1 µL of each culture was transferred into 200 µL of fresh minimal medium with fumarate in a new 96 well plate^16^, and incubated for 2 h at 37 °C with rotary shaking at 700 rpm. An aliquot of 90 µL of each culture was then removed and mixed with 10 µL of ribose solution (10 mM, D-(-)-ribose, Aldrich, USA) in the wells of a new sterile 96-well plate to reach a final ribose concentration of 1 mM. The remaining cell volume (110 µl) in the wells of the previous (uninduced assay) plate were filled with 100 µL of PBS to dilute the cell concentration and measured immediately. The ribose containing assay (induced) was incubated for another 2 h at 37 °C with rotary shaking and then sampled. Assay plate wells were auto-sampled, and values of individual cell forward scatter (FSC) and GFPmut2 fluorescence (FITC-channel) were recorded by a Becton Dickinson Fortessa flow cytometer (LRS FortessaTM, Becton Dickinson, USA). The flow rate was set to 3 µl s^−1^ and the cell density was between 100–1000 cells µL^−1^. Sensitivities for the FSC and the FITC channels were set at 350 V and 676 V, respectively. Recorded data were gated to remove background particles. The mean fluorescence values of the gated uninduced or induced populations were calculated. All experiments were carried out in independent triplicate incubations on the 96-well plate. In all plates, triplicate incubations of *E. coli* strain 4175 (carrying wild-type *rbsB* on pAR3 plus pSYK1) and *E. coli* strain 4497 (with empty vector pSTV28P_AA_mcs plus pSYK1) served as simultaneous positive and negative controls for ribose induction, respectively. Reported fluorescence values are not normalized for plate-to-plate variations.

### Periplasmic space RbsB abundance

The abundance of 25 alanine mutants in the *E. coli* periplasmic space was analyzed using direct peptide mass identification, as described previously^16^. After extraction and separation of the periplasmic protein fraction by SDS-PAGE, proteins in a size region of 28–36 kD were excised from the gel. Subsequently they were digested with trypsin and peptides were separated on an Ultimate 3000 Nano LC System (Dionex), followed by detection in a Thermo Scientific LTQ-Orbitrap XL mass spectrometer (Thermo Fisher Scientific, Waltham, MA). Mass spectra were analyzed by Scaffold Viewer (http://www.proteomesoftware.com/), using protein and peptide identification thresholds of 99.9% and 99.99%, respectively. The minimum number of peptides for identification was 1.

### Crystal structure mutant positioning

Positions of selected Ala-substitutions were visualized on the RbsB ribose-bound (PDB: 2DRI)^22^ and open conformation crystal structure (PDB: 1URP)^21^ using the VMD: visual molecular dynamics viewer^32^.

### Data availability

All relevant data are uploaded as Reimer_Dataset1.xlsx. Ala-substitution mutants are available upon request to the corresponding author.

## Electronic supplementary material

Supplementary information

Dataset 1
