# Supplementary material for: Complete alanine scanning of the Escherichia coli RbsB ribose binding protein reveals residues important for chemoreceptor signaling and periplasmic abundance
Source: Sci Rep. 2017 Aug 15;7:8245. doi: 10.1038/s41598-017-08035-5 (PMC5557919; doi:10.1038/s41598-017-08035-5)
Supplement: Supplementary file 1 — Supplementary information [file 41598_2017_8035_MOESM1_ESM.pdf]

Supplementary Information to

Complete alanine scanning of the *Escherichia coli* RbsB ribose binding protein reveals residues important for chemoreceptor signaling and periplasmic abundance.

Artur Reimer, Vitali Maffenbeier, Manupriyam Dubey, Vladimir Sentchilo, Diogo Tavares, Manuel Hernandez Gil, Siham Beggah, and Jan Roelof van der Meer

Department of Fundamental Microbiology, University of Lausanne, 1015 Lausanne, Switzerland

*corresponding author

J.R. van der Meer

email: janroelof.vandermeer@unil.ch

Supplementary Figures S1-S5

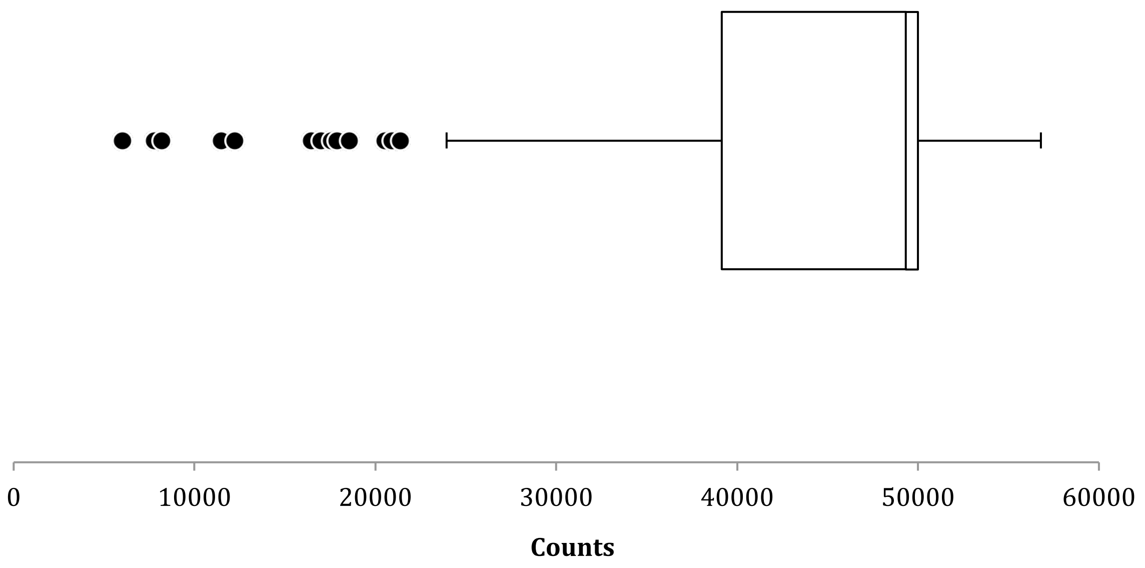

Supplementary Figure 1 Summary cell counts of *E. coli* cultures (in ~200 µl volume) after induction with 1 mM ribose expressing RbsB wild-type or Ala-substitution mutant grown for 4 h in standard fumarate medium as determined by quantitative flow cytometry. Summary counts displayed as box plot (median count = 49333). Outliers are shown as black dots. In detail, these concern RbsB positions 29, 34, 36, 41, 57, 59, 67, 68, 77, 167, 189, and 272.

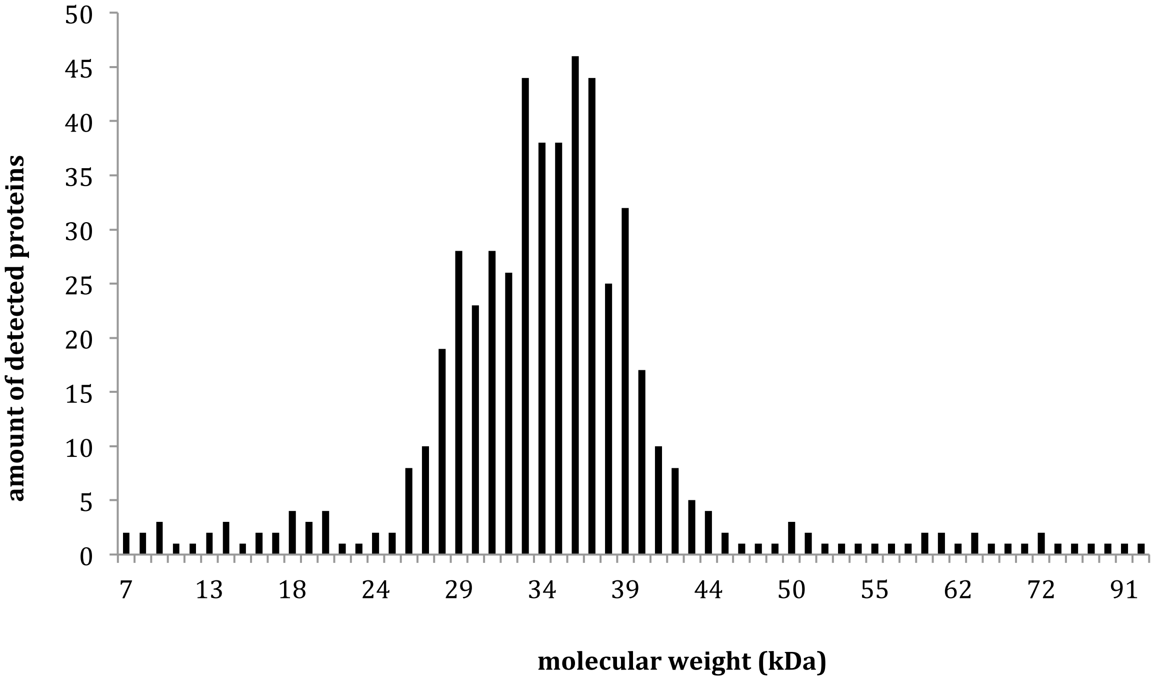

Supplementary Figure 2 Number of detected periplasmic proteins sorted by their predicted molecular weight, confirming the position of the excised gel bands at around 28-40 kDa.

```
Feature 1                 |# ##     |         |         |         |         | #       |         |
2IOY_A         3 TIGLVISTLNNPFFVTLKNGAEEKAKElGYKIIVEDsqndsSKELSNVEDLIQqKVDVLLINPVDSDAVVtAIKEANSKN 82
2FN8_A         4 KMAIVISTLNNPWFVVLAETAKQRAEQlGYEATIFDsqndtAKESAHFDAIIAaGYDAIIFNPTDADGSIaNVKRAKEAG 83
2DRI_A         3 TIALVVSTLNNPFFVSLKDGAQKEADKlGYNLVVLDsqnnpAKELANVQDLTVrGTKILLINPTDSDAVGnAVKMANQAN 82
gi 118047959  39 TIGLVISTLNNPFFVTLRDGAQRAADAaGVTLTIVDaqddsAKMIAGIEDLITkKVNALIINPTDSDAVVpAIQKANEAG 118
gi 148270910  24 VVGLSLSTLNNPFFVTLRDGAVDTAKKlGIDLIVLDaqdnpAKQLNDIEDLIQrGVDLIIINPTDSDAIVsAVESANEAG 103
gi 118443514  35 KVGMVLSTLNNPFFVSMKEGAEKKAKElGCEVLVLDsqndpAKERSNIEDLIQgGVSVLIVNPTDSDAVInSVQVANKAN 114
gi 34498471   43 AVGLAVSTLNNPFFVELRDGAAAEAKKqGVNLITVDaqddpAKQQASVEDLIQkKVSVILINPTDSSAVAnVVKEATSKG 122
gi 23100027   37 KIGLSISTMENPFFVTLHDTIVKEAKEqDMDIVTVNaqddaSVEISGIEDLIQqQVDILLINPTDSAAVSsAVQSANAAG 116
gi 32472547   49 RVAVIVSTLNNPWFVVLAESARDSAIElGYDAVIFDsqndpSKETAHFDNVIAsGYSAVLFNPTDADGSIaNVRRAKEAG 128
gi 108803790  34 TIGLSISTLNNPFFVTLRDGAQRAAKEaGVELIVSDaqndaAQQQDDIQAFITqQVDAILVNPVDSEAVVpAIQAANDAG 113
gi 160941558  67 VIGLAMNTQTNPFFVDVKDGVQKAADEhGIELYITDaqddpTIQMKDVENLITkKPDAIIIDTCDSDAIVsSIEACNEAG 146
gi 164603992  42 SIGFSVSTLNNPFFVTLSQGAKDKAKEeGRDLIVVDagddaAKQTSDIEDLISkNISILIVNPVDSDAVApAVKDAIAKG 121

Feature 1              ## |         |         |         |         |         | #   #   |         |
2IOY_A        83 IPVITIDRsan-ggdVVCHIASDNvkGGEMAAEFIAkal------kgkGNVVELEGIPGasAARDRGKGFDEAiak-ypd 154
2FN8_A        84 IPVFCVDRginarglAVAQIYSDNyyGGVLMGEYFVkflkekypdakeIPYAELLGILSaqPTWDRSNGFHSVvdq-ype 162
2DRI_A        83 IPVITLDRqat-kgeVVSHIASDNvlGGKIAGDYIAkka------gegAKVIELQGIAGtsAARERGEGFQQAvaa--hk 153
gi 118047959 119 IPVFTVDRgan-ggtVVSHIASDNvaGGRMAAEFLCnal------ggkGKVVELQGIAGtsAARDRGQGFNDYmassckg 191
gi 148270910 104 IPVITVDRasn-ggkVVCHIASDNveGGRMAARYIAkvl------ngkGKVVELVGIPGtsAARDRGKGFEEElak-ypg 175
gi 118443514 115 IPVITVDRqan-ggkVISHIASDNtkGGELAGKYIIdtlk----dkkdIKVVELQGIPGasATRERGAGFHNIidk-knn 188
gi 34498471  123 IKVVSLDRsvn-gaeVSAHIASDNiaGGVMAGKYLLdkl------ggkGRIVELEGIAGssAARERGEGFHQVvdk-kdg 194
gi 23100027  117 IPVITIDRsad-egeVETLITSDNisGGEMAAEFIAdtv------genANVVELQGIAGasATRERGEGFHNIae---er 186
gi 32472547  129 VPVFCMDReinatdaAVSQILSDNysGCVAIGQHFVkev------gesGEYAELLGLVGdnNTRNRSDGFHSVvdr-ype 201
gi 108803790 114 IPVIALDRgaa-ggeIETLIASDNveGGRMAARELIrlv-------gsGPVAQLEGIPGtsAARDRGKGFEEVieg-qda 184
gi 160941558 147 IPVFTMDRean-ggeVISHIGYDAikSGRMAGQYLVdtl------ggkGKIVEIQGIMGtnVAQNRSQGFNEVmkd-npd 218
gi 164603992 122 IKVISLDRavn-gvdVDCSIASDNveGAKMATEYLVslv------gkdAKVVELEGTSGssATIDRGTGFHKVad---eq 191

Feature 1                 |#        |         |      #  |         |         |  #      |         |
2IOY_A       155 IKIVAKQAADFDRSKGLSVMENILQaqpkidAVFAQNDEMALGAIKAIEAAnr-qgIIVVGFDGteDALKAIk-eGKMAA 232
2FN8_A       163 FKMVAQQSAEFDRDTAYKVTEQILQahpeikAIWCGNDAMALGAMKACEAAgr-tdIYIFGFDGaeDVINAIkegKQIVA 241
2DRI_A       154 FNVLASQPADFDRIKGLNVMQNLLTahpdvqAVFAQNDEMALGALRALQTAgk-sdVMVVGFDGtpDGEKAVn-dGKLAA 231
gi 118047959 192 VEIVAQQTADFNRDRGLRVFENILQaqpeinGVFAHNDEMILGAIQAAEAAgr-sgIVFVGFDAidDATKAVq-eGKLAA 269
gi 148270910 176 LQLVAKQTANFNRAEGLTVMENILEahpdidAVFAQNDEMALGAIEAIKAAgklddIIVVGFDAipDAIEAIk-kGEMEA 254
gi 118443514 189 VKVVSSQAANFDRAQGLSVMENIIQaqsdfdAVFAHNDEMALGAAKALKTAnr--nVMVIGFDGdeDARAAId-kKEMVA 265
gi 34498471  195 VKLLAKQPADFDRAKGLSVMENIIQgnkdiqGVFAHNDEMALGAVKAIQAAgl-knVVVVGFDAtpDAVAAVk-gGTLSA 272
gi 23100027  187 LNVLVSQPANFDRTEGLTVMENTLQgysgidAVFAHNDEMALGAIEAIKASge--dIVVVGFDGvdDAIAAVe-aGELTA 263
gi 32472547  202 LKMVAQQSADFDRAKALEVMEAILQanpdikAVFCGNDAMAMGAYQALLAAgkaeqVKIFGFDGadDVVAMIq-eGKIVA 280
gi 108803790 185 VRLVASQPANFDRAQGLNVTQNILQahpeikGIFAQNDEMALGAVRALGERag-teVKIVGFDAieDALKAIr-dGRMNA 262
gi 160941558 219 MEIVACQVADFDRAKGMSVMENILQanpeidGLYAANDEMLLGALEAMEAAgrtdeIVKIGCDAidDTLDAMk-aGKVDA 297
gi 164603992 192 LNVVSSQTANFNRSEGMTVMENMLQsnsdikGVFAHNDEMALGAVEAIGNKd----IVVVGFDStdDALAAIk-kGKMAA 266

Feature 1           #     |         |         |
2IOY_A       233 TIAQQpALMGSLGVEMADkylkg-ekipNFIPAELKLIT 270 Thermoanaerobacter tengcongensis
2FN8_A       242 TIMQFpKLMARLAVEWADqylrgersfpEIVPVTVELVT 280 Thermotoga maritima
2DRI_A       232 TIAQLpDQIGAKGVETADkvlkg-ekvqAKYPVDLKLVV 269 Escherichia coli K12 RbsB
gi 118047959 270 TVAQQpALMGQTAVETVVkylkg-dkveSSIPVPLSLVT 307 Chloroflexus aggregans DSM 9485
gi 148270910 255 TIAQQpYLMGQLAVTKAFeylt---tgtVFFPVELKLVT 290 Thermotoga petrophila RKU-1
gi 118443514 266 TVAQQpVLMGEAAVDNAMkilng-enipRQIPVKLTLVT 303 Clostridium novyi NT
gi 34498471  273 TVQQQpALIGQYGVQTAKkladg-qkvdKFIPVPLNLVH 310 Chromobacterium violaceaum ATCC12472
gi 23100027  264 TIAQQpNLMGEEAIATVKavlng-nevdEVIPVPLELKT 301 Oceanobacillus iheyensis HTE831
gi 32472547  281 TGMQFpKLMAKTAAEYADqylkgdrdllQKVPVAVELVH 319 Rhodopirellula baltica SH 1
gi 108803790 263 TIAQQpAKMGSLGVRNAIkvieg-esvpKNIPVEVRLVT 300 Rubrobacter xylanophilus DSM 9941
gi 160941558 298 TIAEPpFFLGKAILNTAYdyleg-kqvePYVILDNQLVT 335 Clostridium bolteae ATCC BAA-613
gi 164603992 267 TVAQKpDLMGATAVETAIkiing-eaveKSIPVEVELVT 304 Intestinibacter bartlettii DSM16795
```

Supplementary Figure S3 Alignment of the top members of the cd06323:PBP1_ribose_binding conserved protein domain family. cd06323 is a member of the superfamily cI10011. The first three aligned proteins are structure determined with ribose bound. The Feature is based on observed ribose contacts in the protein structure. Alignment produced by CDD [1]. Residues in the *E. coli* RbsB 2DRI_A sequence highlighted in gold: Ala-substitution aborts induction (see Table 1); in magenta: Ala-substitution diminishes induction; in red: Ala-substitutions not obtained.

```
ATATACATATGAACATGAAAAAACTGGCTACCCTGGTTTCCGCTGTTGCGCTAAGCGCCACCGTCAGT
         M  N  M  K  K  L  A  T  L  V  S  A  V  A  L  S  A  T  V  S
GCGAATGCCATGGCAGCGGACACCATCGCGCTGGTGGTCTCCACGCTTAACAACCCGTTCTTTGTATC
 A  N  A  M  A  A  D  T  I  A  L  V  V  S  T  L  N  N  P  F  F  V  S
GCTGAAAGATGGCGCGCAGAAAGAGGCGGATAAACTTGGCTATAACCTGGTGGTGCTGGACTCCCAGA
  L  K  D  G  A  Q  K  E  A  D  K  L  G  Y  N  L  V  V  L  D  S  Q
ACAACCCGGCGAAAGAGCTGGCGAACGTGCAGGACTTAACCGTTCGCGGCACAAAAATTCTGCTGATT
N  N  P  A  K  E  L  A  N  V  Q  D  L  T  V  R  G  T  K  I  L  L  I
AACCCGACCGACTCCGACGCAGTGGGTAATGCTGTGAAGATGGCTAACCAGGCGAACATCCCGGTTAT
 N  P  T  D  S  D  A  V  G  N  A  V  K  M  A  N  Q  A  N  I  P  V  I
CACTCTTGACCGCCAGGCAACGAAAGGTGAAGTGGTGAGCCACATTGCTTCTGATAACGTACTGGGCG
  T  L  D  R  Q  A  T  K  G  E  V  V  S  H  I  A  S  D  N  V  L  G
GCAAAATCGCTGGTGATTACATCGCGAAGAAAGCGGGTGAAGGTGCCAAAGTTATCGAGCTGCAAGGC
G  K  I  A  G  D  Y  I  A  K  K  A  G  E  G  A  K  V  I  E  L  Q  G
ATTGCTGGTACATCCGCAGCCCGTGAACGTGGCGAAGGCTTCCAGCAGGCCGTTGCTGCTCACAAGTT
 I  A  G  T  S  A  A  R  E  R  G  E  G  F  Q  Q  A  V  A  A  H  K  F
TAATGTTCTTGCCAGCCAGCCAGCAGATTTTGATCGCATTAAAGGTTTGAACGTAATGCAGAACCTGT
  N  V  L  A  S  Q  P  A  D  F  D  R  I  K  G  L  N  V  M  Q  N  L
TGACCGCTCATCCGGATGTTCAGGCTGTATTCGCGCAGAATGATGAAATGGCGCTGGGCGCGCTGCGC
L  T  A  H  P  D  V  Q  A  V  F  A  Q  N  D  E  M  A  L  G  A  L  R
GCACTGCAAACTGCCGGTAAATCGGATGTGATGGTCGTCGGATTTGACGGTACACCGGATGGCGAAAA
 A  L  Q  T  A  G  K  S  D  V  M  V  V  G  F  D  G  T  P  D  G  E  K
AGCGGTGAATGATGGCAAACTAGCAGCGACTATCGCTCAGCTACCCGATCAGATTGGCGCGAAAGGCG
  A  V  N  D  G  K  L  A  A  T  I  A  Q  L  P  D  Q  I  G  A  K  G
TCGAAACCGCAGATAAAGTGCTGAAAGGCGAGAAAGTTCAGGCTAAGTATCCGGTTGATCTGAAACTG
V  E  T  A  D  K  V  L  K  G  E  K  V  Q  A  K  Y  P  V  D  L  K  L
GTTGTTAAGCAATTGCACCACCACCACCACCACTGACAATTGTAAGTCGACAGGCATGCAAGCTTGGC
 V  V  K  Q  L  H  H  H  H  H  H STOP
```

Supplementary Figure 4 Exemplary sequence for mutant K1A of RbsB (numbering starting at the mature protein without signal sequence). Start codon (yellow), place of mutation (green), first amino acid of mature protein (red), and stop codons (blue) are highlighted.

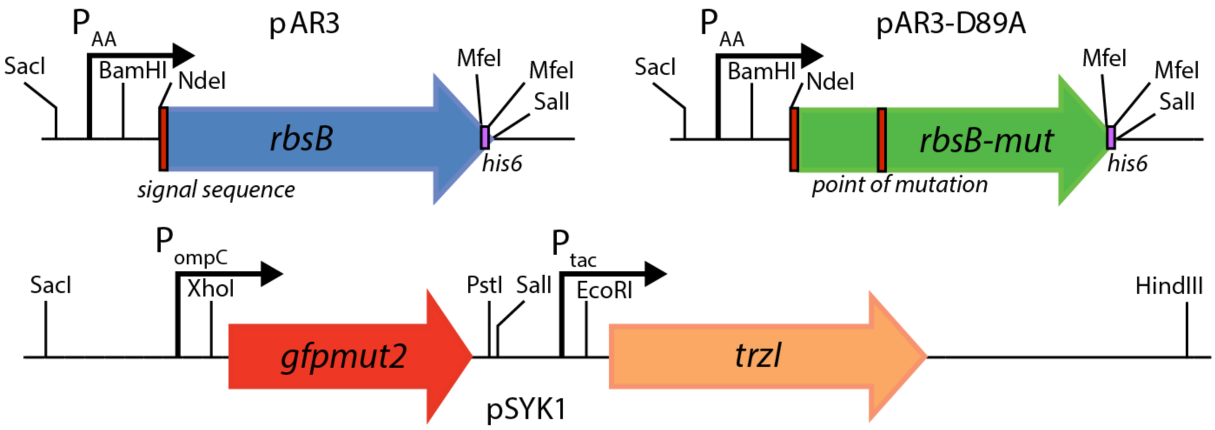

Supplementary Figure 5 Relevant vectors and plasmid constructions. *rbsB* or *rbsB-mut* (here exemplary D89A) with original *rbsB* periplasmic export signal sequence and 3'-hexahistidine tag under transcriptional control of the weak constitutive P_{AA} promoter [2]. Plasmid pSYK1 with *gfpmut2* under the *ompC* promoter control and *trzI* under control of P_{tac} (note that pSYK1 carries the *lacI^q* gene) cotransformed in the same strain to provide the signaling chain.

References

1. Marchler-Bauer, A. *et al.* CDD: NCBI's conserved domain database. *Nucleic Acids Res* **43**, D222-226 (2015).
2. Alper, H., Fischer, C., Nevoigt, E. & Stephanopoulos, G. Tuning genetic control through promoter engineering. *Proc Natl Acad Sci U S A* **102**, 12678-12683 (2005).
